# Determinants of riverine migration success by Atlantic salmon (*Salmo salar*) smolts from rivers across the UK and Ireland

**DOI:** 10.1111/jfb.15884

**Published:** 2024-08-12

**Authors:** Jessica R. Rodger, Jemma Guthrie, Hannele M. Honkanen, Angus J. Lothian, Jessie Lilly, Marcus Walters, Richie Miller, Lorraine Hawkins, Al Reeve, Jamie Ribbens, Jim Henderson, Debbie Parke, Amy Green, Brian A. Shields, Philip Ramsden, Melanie Fletcher, Alan Kettle‐White, Brian Shaw, Stephen Burns, Robert Laughton, Chris Conroy, Chris Daphne, Keith Williams, Sean Robertson, Colin W. Bean, Diego del Villar, Catherine Waters, Robert Rosell, Deirdre Cotter, Melanie Smith, Niall Ó. Maoiléidigh, Richard Kennedy, Colin E. Adams

**Affiliations:** ^1^ Scottish Centre for Ecology and the Natural Environment, School of Biodiversity, One Health and Veterinary Medicine University of Glasgow Glasgow UK; ^2^ Atlantic Salmon Trust Perth UK; ^3^ Northwest Atlantic Fisheries Centre St Johns Newfoundland Canada; ^4^ The Deveron Bogie and Isla Rivers Charitable Trust Avochie UK; ^5^ Dee District Salmon Fishery Board Aboyne UK; ^6^ Galloway Fisheries Trust Newton Stewart UK; ^7^ The Nith Catchment Fishery Trust and Nith District Salmon Fishery Board Dumfries UK; ^8^ Environment Agency Ghyll Mount Penrith UK; ^9^ Natural England Penrith UK; ^10^ Argyll Fisheries Trust Inveraray UK; ^11^ The Ness District Salmon Fishery Board, Beauly House Dochfour Business Centre Inverness UK; ^12^ The Spey Fisheries Board Aberlour UK; ^13^ The Findhorn Nairn and Lossie Rivers Trust Forres UK; ^14^ The Kyle of Sutherland Fisheries Ardgay UK; ^15^ Loughs Agency Londonderry UK; ^16^ Marine Institute Newport Ireland; ^17^ Agri‐food and Biosciences Institute River Bush Salmon Station Bushmills UK

**Keywords:** acoustic telemetry, fresh water, migration, *Salmo salar*, spatial variation, temporal variation

## Abstract

There is some evidence that the river migration success of Atlantic salmon smolts, on their first migration to sea, varies both spatially and temporally. However, we have only a poor understanding of what may be driving this variation. In this study, we used acoustic telemetry to quantify the spatial and temporal variations in river migration success in Atlantic salmon smolts on their first migration to sea. In total 4120 Atlantic salmon smolts migrating through 22 rivers in Scotland, England, Ireland, and Northern Ireland over multiple years were included in the study. Individuals were defined as successful migrants if detected leaving the river to enter marine waters. The results show significant temporal (up to 4 years) and spatial (river) variations in migration success, with overall between‐river migration success varying from 3.4% to 97.0% and between years from 3.4% and 61.0%. Temporal variation in migration success was river specific, with some rivers being more temporally stable (exhibiting little variation between years) than others. Across all rivers and years, individual migration success was predicted positively by body condition and negatively by tag burden. The rate of migration success for a population (migration success standardized to a common river distance [proportion km^−1^]) was predicted by a number of environmental factors. The proportion of river catchment that comprised wetland and woodland positively predicted migration success, whereas the proportion of grassland and peatland in a catchment negatively predicted the rate of migration success. Although the mechanisms through which these effects may be operating were not directly examined in this study, we discuss some potential routes through which they may occur.

## 1 INTRODUCTION

The costs of undertaking a long‐distance migration are multiple and significant. They include the obvious energetic and time costs associated with moving between habitats (Bonte et al., 2012; Dingle, 1996) but also the potential costs associated with the risk of navigational error and the uncertainties of achieving benefits from that migration (see Adams et al., 2022, and references therein). Where it has been measured, it is perhaps not surprising that mortality rates are frequently higher during periods of migration than they are at other stages in the life cycle of an animal (Cresswell et al., 2011; Guillemain et al., 2010). Thus, for those species that migrate, this phase in the life cycle has the potential to have a disproportionate effect on the dynamics of the population.

The Atlantic salmon, *Salmo salar*, typically undertakes a long‐distance migration from its natal rivers to the open ocean (Thorstad et al., 2012). Higher productivity and growth opportunity in the marine habitats to which they migrate allow for a more rapid growth than could be achieved in fresh water, which in turn confers higher reproductive output for both females and males of this species (Hutchings & Myers, 1994; Kinnison et al., 2003; Sandlund et al., 2014). However, as with other species, migration is risky for Atlantic salmon, and the proportion of migrants returning to fresh water to spawn rarely exceeds 10% and is frequently much lower (Friedland et al., 2000; Potter & Crozier, 2000). The emergence and subsequent development of acoustic telemetry techniques have allowed for more precise spatial and temporal insights into migration behavior. One important development, which has been facilitated by these technological advances, is to look beyond overall migration return rates of adults and partition migration success at finer spatial and temporal scales. Thus, it is possible to match specific catchments, habitats, environments, life events, or their transitions to the migration success of an individual (Drenner et al., 2012; Hussey et al., 2015).

There is evidence that the migration success (i.e., the successful passage from any one spatial audit point to another) of Atlantic salmon smolts migrating to sea varies between rivers. For example, migration success of salmon smolts from two tributaries of the River Clyde, Scotland, was measured as 22% and 91% in a single year (Lilly et al., 2022). Similarly, Chaput et al. (2019) showed that the probability of salmon smolt survival during their riverine migration ranged from 20% to 100% over 14 years, in four rivers draining into the Gulf of St. Lawrence (Canada). Thorstad et al. (2012) reviewed the available literature and showed broad, habitat‐specific (in this case, river, estuarine, and coastal marine habitats) differences in migration success for Atlantic salmon smolts on their first migration to sea. For example, a number of studies have shown that the migration success of salmon smolts through lakes is frequently lower than through rivers (Aarestrup et al., 1999; Honkanen et al., 2018, 2021; Kennedy et al., 2018; Thorpe et al., 1981). In addition, migration success of salmon smolts in a river catchment modified by barriers is often lower in comparison to unobstructed sections of the river (Huusko et al., 2018). Thus, in addition to broad, habitat‐specific differences in migration success, there appears to be considerable variation in migration success among salmon smolts migrating through apparently similar habitat types (Thorstad et al., 2012).

Where it has been measured, there is also evidence of temporal variation in migration success of salmon smolts (Chaput et al., 2019). For example, Flávio et al. (2021) demonstrated temporal variation in the overall survival of salmon smolts during migration, varying between 30% and 55% over the 3 years of the study in the River Minho, Spain. Despite the evidence of some spatial and temporal differences in migration success of Atlantic salmon smolts as they migrate to sea, to date we have only a poor understanding of what factors may be driving this variation. An obvious group of candidate drivers of riverine migration success in Atlantic salmon smolts are the environmental characteristics of the catchment. Therefore, in this study, we attempt to look at potential broad, landscape‐scale effects (specifically land‐use and underlying geology characteristics) on the rate of migration success between different river populations. For individual fish, migration success may be linked with intrinsic factors, for example, body condition (Antonsson et al., 2010; Armstrong et al., 2018). In addition, some concern has been expressed about the impact of acoustic tags on normal migration behavior (see Lennox et al., 2022; Newton et al., 2016). Thus, in this study, we also examined the potential effects of body size, body condition, and tag burden on successful migration of smolts through rivers.

Here we combine telemetry studies on Atlantic salmon smolt migration in 22 rivers in Scotland, England, Ireland, and Northern Ireland between 2019 and 2022. We use the data to describe the nature of the spatial and temporal variations in migration success of salmon smolts as they migrate downstream in rivers during the spring and examine the environmental factors that may be driving that variation. Specifically, we

- quantify the spatial variation in the migration success of salmon smolts across rivers, and
- quantify the temporal (between‐year) variation in smolt migration success.

We also test the following null hypotheses:

- Individual smolt migration success cannot be predicted by body condition, body size, or tag burden.
- Population‐level migration success is not influenced by landscape‐scale land‐use and geological features of a river catchment.

## 2 METHODS

### 2.1 Study area

**FIGURE 1:**
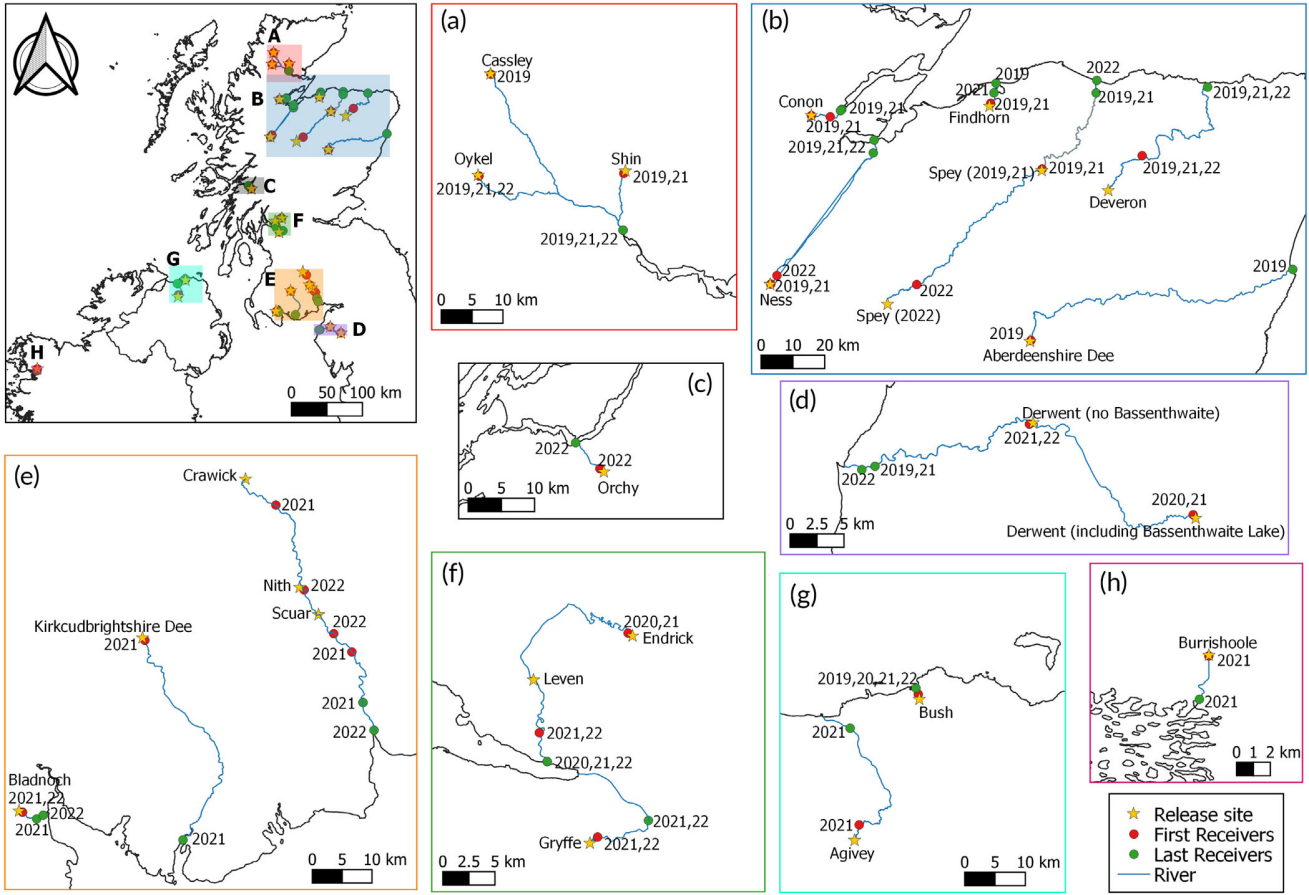
Location of the 22 study rivers and of the acoustic receivers included in this study. Stars indicate release sites (see Table 1 for details), red points indicate the most upstream receivers in the study, and green points are the most downstream receivers in the study. Expanded detail provided in: a) for the Rivers Shin, Cassley and Oykel, b) for the Rivers Conon, Ness, Findhorn, Spey, Deveron and Aberdeenshire Dee, c) for the River Orchy, d) for the River Derwent, e) for the Rivers Bladnoch, Kirkcubrightshire Dee and Nith, f) for the Rivers Endrick and Gryffe, g) for the Rivers Bush and Agivey ) for the River Burrishoole. Note that for some rivers, receiver placement varied between years; therefore, the year a receiver was deployed has been included next to the receiver (for more details see Table S1).

The overall objectives of the nine acoustic telemetry projects included in this study were somewhat different, but all nine projects included an element that investigated the downstream migration of Atlantic salmon smolts during their spring migration. The nine projects encompassed 22 rivers across Scotland, England, Ireland, and Northern Ireland (Figure 1; Table 1). These 22 rivers represented a range of geologies and landscape‐scale characteristics with variation between countries, within countries, and often between adjacent rivers.

**TABLE 1:** The location (river) and year of each telemetry project included in this study, the latitude and longitude of the fish release site, the tag type used, the nominal delay between transmissions, the number of smolts tagged, the range of tagging dates and mean fish length, mass, body condition (calculated as the residual for that fish drawn from a regression of log mass on log length for all fish tagged), and tag burden (defined as the ratio of tag mass to fish mass in air).

River	Year	Latitude	Longitude	Tag type	Number tagged	Date tagged	Mean fork length (mm ± SD)	Mean mass (g ± SD)	Mean body condition (± SD)	Mean tag burden (% ± SD)
Aberdeenshire Dee	2019	56.933	−3.420	V5‐1x	105	01/04–28/04	133 (±7.32)	24.3 (±4.05)	0.034 (±0.055)	2.7 (±0.4)
Agivey	2021	54.988	−6.666	V7‐2x	41	20/04	151 (±9.77)	36.0 (±7.49)	0.030 (±0.101)	4.4 (±1.0)
Bladnoch	2021	54.867	−4.498	V7‐2x	127	20/04–15/05	142 (±7.28)	29.1 (±4.67)	0.022 (±0.053)	5.3 (±0.8)
Bladnoch	2022	54.867	−4.498	V7‐2x	100	25/04–03/05	142 (±6.31)	29.1 (±4.14)	0.011 (±0.071)	5.6 (±0.7)
Burrishoole	2021	53.914	−9.571	V7‐2x, V8‐4x, V7D‐2x	85	05/05–11/05	184 (±23.91)	71.5 (±29.50)	0.074 (±0.108)	3.0 (±1.2)
Bush	2019	55.203	−6.523	V7‐4x	49	18/04–02/05	157 (±8.82)	38.4 (±5.36)	0.002 (±0.078)	4.8 (±0.6)
Bush	2020	55.203	−6.523	V7‐4x	100	15/04–13/05	169 (±11.26)	45.5 (±5.64)	−0.047 (±0.147)	4.0 (±0.5)
Bush	2021	55.203	−6.523	V7‐4x	80	13/04–26/04	169 (±9.85)	48.5 (±9.89)	0.007 (±0.076)	3.8 (±0.6)
Bush	2022	55.203	−6.523	V7‐4x	100	05/04–30/04	171 (±8.51)	52.1 (±7.70)	0.051 (±0.081)	3.5 (±0.5)
Cassley	2021	58.143	−4.780	V7‐2x	47	26/04–04/05	140 (±6.28)	25.2 (±3.91)	−0.090 (±0.082)	6.1 (±0.9)
Conon	2019	57.553	−4.593	V7‐2x	99	14/04–07/05	139 (±5.39)	26.6 (±3.35)	−0.016 (±0.044)	5.7 (±0.7)
Conon	2021	57.553	−4.593	V7‐2x	100	16/04–07/05	145 (±8.56)	30.0 (±5.53)	−0.028 (±0.061)	5.1 (±0.8)
Crawick	2021	55.378	−3.931	V7‐2x	49	15/04–23/04	138 (±5.17)	26.8 (±2.76)	0.021 (±0.063)	5.7 (±0.6)
Derwent (released at trapping site)	2020	54.611	−3.062	V7‐2x	100	01/05–03/05	139 (±6.49)	27.9 (±4.25)	0.030 (±0.096)	5.5 (±0.8)
Derwent (released at trapping site)	2021	54.611	−3.062	V7‐2x	93	16/04–05/05	142 (±8.39)	29.5 (±5.56)	0.026 (±0.059)	5.5 (±1.0)
Derwent (transported)	2021	54.688	−3.298	V7‐2x	57	20/04–04/05	141 (±7.63)	29.7 (±5.11)	0.057 (±0.048)	5.5 (±0.9)
Derwent (transported)	2022	54.688	−3.298	V7‐2x	115	05/05–25/05	139 (±6.65)	27.4 (±4.14)	0.027 (±0.055)	5.6 (±0.7)
Deveron	2019	57.363	−3.028	V7‐2x, V7D‐2x	100	13/04–30/04	134 (±5.04)	23.5 (±2.66)	−0.013 (±0.058)	6.7 (±0.6)
Deveron	2021	57.363	−3.028	V7‐2x, V7D‐2x	100	13/04–30/04	137 (±4.83)	25.1 (±2.96)	−0.022 (±0.060)	6.0 (±0.7)
Deveron	2022	57.363	−3.028	V7‐2x, V6‐2x	100	04/04–30/04	137 (±6.88)	25.3 (±3.94)	−0.018 (±0.069)	4.8 (±1.3)
Endrick	2020	56.049	−4.440	V7‐2x	158	13/04–20/04	146 (±17.73)	32.2 (±15.07)	−0.005 (±0.098)	5.1 (±1.2)
Endrick	2021	56.049	−4.440	V7‐2x	99	15/04–04/05	143 (±10.12)	29.5 (±6.33)	0.010 (±0.069)	5.3 (±1.0)
Findhorn	2019	57.595	−3.659	V7‐2x	100	14/04–16/05	135 (±6.05)	23.6 (±3.58)	−0.051 (±0.052)	6.5 (±0.9)
Findhorn	2021	57.595	−3.659	V7‐2x	93	13/04–02/05	137 (±6.62)	23.7 (±3.16)	−0.082 (±0.062)	6.4 (±0.7)
Kirkcudbrightshire Dee	2021	55.134	−4.193	V7‐2x	50	17/04–23/05	148 (±11.23)	32.0 (±6.88)	−0.028 (±0.054)	4.9 (±1.2)
Gryffe	2021	55.869	−4.494	V7‐2x	101	14/04–26/04	149 (±10.17)	34.0 (±6.69)	0.024 (±0.076)	4.6 (±0.9)
Gryffe	2022	55.869	−4.494	V7‐2x	122	12/04–24/04	142 (±6.51)	28.7 (±4.2)	−0.001 (±0.084)	5.3 (±0.9)
Leven	2021	56.009	−4.590	V7‐2x	46	13/04–01/05	148 (±10.35)	32.6 (±6.75)	−0.001 (±0.119)	4.8 (±0.9)
Leven	2022	56.009	−4.590	V7‐2x	178	14/04–06/05	143 (±7.52)	30.1 (±4.84)	0.027 (±0.070)	5.1 (±0.8)
Ness	2019	57.071	−4.773	V7‐2x, V7D‐2x	100	12/04–26/04	140 (±10.91)	28.8 (±6.73)	0.035 (±0.063)	5.6 (±0.9)
Ness	2021	57.071	−4.773	V7‐2x	120	06/04–30/04	140 (±7.88)	28.2 (±5.10)	0.027 (±0.065)	5.5 (±0.9)
Ness	2022	57.071	−4.773	V6‐2x, V7D‐2x	74	05/04–23/04	142 (±9.20)	30.3 (±6.12)	0.057 (±0.048)	4.1 (±1.2)
Nith	2021	55.176	−3.729	V7‐2x	130	23/04–06/05	148 (±10.85)	33.7 (±7.10)	0.004 (±0.074)	4.8 (±1.0)
Nith	2022	55.176	−3.729	V7‐2x	100	18/04–30/04	146 (±11.65)	31.9 (±8.23)	0.014 (±0.052)	5.3 (±1.2)
Orchy	2022	56.404	−5.157	V7‐2x	26	29/04–01/05	143 (±8.28)	27.7 (±5.18)	−0.052 (±0.076)	5.9 (±0.9)
Oykel	2019	57.994	−4.803	V7‐2x	149	11/04–03/05	137 (±7.24)	25.3 (±3.94)	−0.017 (±0.058)	6.1 (±0.8)
Oykel	2021	57.994	−4.803	V7‐2x	100	16/04–27/04	140 (±7.47)	25.7 (±4.28)	−0.066 (±0.043)	6.0 (±0.9)
Oykel	2022	57.994	−4.803	V6‐2x	50	09/04–22/04	142 (±9.06)	27.9 (±5.91)	−0.028 (±0.062)	3.3 (±0.5)
Scaur	2022	55.217	−3.781	V7‐2x	75	14/04–23/04	140 (±8.71)	27.7 (±5.92)	0.014 (±0.041)	6.0 (±0.9)
Shin	2019	58.010	−4.399	V7‐2x	100	11/04–27/04	136 (±4.83)	24.2 (±2.50)	−0.038 (±0.054)	6.2 (±0.6)
Shin	2021	58.010	−4.399	V7‐2x	100	16/04–24/04	140 (±6.63)	25.7 (±3.60)	−0.067 (±0.058)	5.9 (±0.8)
Spey	2019	57.416	−3.377	V7‐2x	150	13/04–02/05	134 (±3.59)	24.0 (±2.46)	−0.010 (±0.059)	6.3 (±0.6)
Spey	2021	57.416	−3.377	V7‐2x	100	14/04–02/05	135 (±4.10)	24.2 (±2.37)	−0.022 (±0.050)	6.2 (±0.6)
Spey	2022	57.416	−3.377	V7D‐2x, V6‐2x	126	04/04–07/05	138 (±5.98)	27.1 (±3.90)	0.050 (±0.218)	3.9 (±0.9)

### 2.2 Ethics statement

All methods and procedures for this study complied with animal welfare laws, guidelines, and policies and were conducted under license by the appropriate national authorities (UK Home Office Licenses: PP0483054 and PPL 2869; Irish Health Products Regulatory Authority License: AE19121/P003).

### 2.3 Fish capture and tagging

Salmon smolts were captured at the 22 study sites between April and May in at least 1 year between 2019 and 2022 (Table 1) using 1.5‐m diameter rotary screw traps, fyke nets, and angling (River Agivey smolts only [*N* = 41]) and wolf‐type traps.

Following standard surgical tagging methods (see Lilly et al. 2022), captured salmon smolts were anaesthetized and then measured for fork length (mm) and mass (g). Salmon smolts with a fork length ≥130 mm and mass ≥20 g were selected for tagging using V7‐2x acoustic tags (Innovasea, Canada), which were used to tag the majority of fish in this study (Table 1). At some sites V5‐2x (minimum fish fork length for tagging with this tag was 120 mm), V6‐2x (minimum fork length of tagged fish 130 mm), V7‐4x and V7D‐2x (minimum fork length of tagged fish 175 mm), and V8‐4x (minimum fork length of tagged fish 175 mm) acoustic tags (Table 1; see Table S2 for tag specifications) were used. Following anesthesia and measurement, fish were placed on a damp v‐notched sponge, and a small incision was made anterior to the pelvic girdle and into the abdominal cavity. An acoustic tag was inserted into the cavity through the incision, and the incision was closed with two interrupted sutures using modified surgeon's knots. Smolts were then placed in aerated river water and/or perforated holding containers in the river. Smolts were held for a minimum of 1 h (fish tagged on the River Burrishoole were held overnight and released the following day) until fully recovered, at which point the smolts were released. Smolts were either released immediately downstream of the trapping site or were transported further downstream before being released at another location on the River Shin (in 2019, 2021 & 2022), the River Conon (in 2019, 2021 & 2022), River Derwent (in 2021 & 2022) and the River Endrick (in 2021 & 2022) (Table 1).

### 2.4 Acoustic receiver deployment

For this study, two receivers were deployed in each river in each year: one below the release site to determine the number of smolts that entered the study system and one at the mouth of each river to determine the number of smolts that successfully left fresh water and entered estuarine or marine waters. For some rivers, receiver placement varied between years (Figure 1; Table S1). A total of 88 acoustic receivers (Innovasea, models: VR2W and VR2Tx) were deployed in this study (Figure 1; Table S1).

### 2.5 Environmental data

Data for fixed catchment‐specific variables were collated for each river catchment, and data for variables that varied temporally were collated for each river in each year of the study. River distance between the first and last receivers in each river was calculated using the QNEAT3 plugin (Raffler, 2023) in QGIS (QGIS Development Team, 2023). To test for potential effects of river impoundment on migration success (Huusko et al., 2018), the number of artificial barriers to fish movement between the first and last receivers was extracted from datasets comprising the SEPA “Obstacles to Fish Passage” dataset (SEPA WMS, 2018), the Environment Agency, “Fish and Eel Migration Barrier” database (Environment Agency, 2019), and the Agivey Anglers Association (McLaughlin, 2020). To calculate the gradient of each river, the elevation of all receivers was obtained from an integrated hydrological digital terrain model (CEH, 2016). Gradient was then calculated as the ratio of the elevation difference to the horizontal river distance between the first and last receivers in each river. To quantify river discharge for each river for each year, time series of daily mean discharge was extracted from gauging stations in the UK Hydrometric Network (CEH, 2023) and from the Environmental Protection Agency Hydrotool (EPA, 2023). These data were used to construct river‐specific flow duration curves using the *hydroTSM* package (Zambrano‐Bigiarini, 2024). A flow duration curve describes the flow characteristics of that river and comprises the proportion of time that any given river discharge is exceeded (percentage exceedance; usually expressed in units such as Q95 and Q50) for that specific river. In this case a cubic spline was fitted to define the relationship between river flow and percentage exceedance. The *uniroot* function in R was used to find the *Q*‐value (percentage exceedance) for any observed river discharge. Percentage exceedance thus provides a standardized measure of the magnitude of a discharge independent of the overall size of the river. Therefore, discharge was calculated for each river and each year as the average (mean) of the daily mean discharge, during the time period between the day that the first smolt was tagged and the day the last smolt was detected on the most downstream receiver. Mean daily discharge was then used to determine the equivalent percentage exceedance specific to that river and specific to the migration period of that fish.

Landscape‐scale, catchment environmental variables comprising land use and geology were calculated based on the whole of each river catchment. Catchment areas were obtained from the HydroBASINS dataset (Lehner & Grill, 2013). The percentage of catchment land cover in five ecologically relevant categories (grassland, agricultural land, woodland, wetland, and urban areas) was drawn from the UKCEH Land Cover Map 2021 for UK catchments (Marston et al., 2022) and from CORINE land cover data for Irish catchments (European Environment Agency, 2018). The dominant bedrock type (igneous, metamorphic, or sedimentary) in each catchment was extracted from the British Geological Survey and Geological Survey Ireland (British Geological Survey, 2023; Geological Survey Ireland, 2013). The proportion of peat in the superficial geology of each catchment was determined using the British Geological Survey and Geological Survey Ireland (British Geological Survey, 2023; Geological Survey Ireland, 2013).

### 2.6 Statistical approach

All analyses were carried out in R, version 4.2.1 (R Core Team, 2022). Raw tag detection data were filtered to remove duplicate detections, false detections from outside the time period of the study and transmitter codes not used in the study.

Summary statistics comprising mean fork length (mm), mass (g), body condition, and tag burden were calculated for smolts tagged at each river (Table 1). Body condition was determined as the residual for that fish drawn from a regression of log mass on log length for all fish tagged, thus providing a measure of mass independent of fish length. Where a fish was tagged and either mass or length was not recorded, that fish was removed from further analysis of individual migration success (*n* = 23). Tag burden was defined as the ratio of the mass of the acoustic tag (g) to the mass of the fish (g) in air, expressed as a percentage. Both body condition and tag burden were used in further analysis.

#### 2.6.1 Determinants of individual river migration success

To determine whether individual migration success was affected by characteristics of the individual fish, generalized linear mixed effects models, with a binomial distribution, were constructed using freshwater migration success of individual fish as a response variable (unsuccessful or successful) and tag burden and body condition as continuous explanatory variables, with river and year included as random effects.

The variance inflation factor of each explanatory variable in the most complex model was calculated using the *car* package (Fox & Weisberg, 2019) to determine whether multicollinearity was present between explanatory variables. Variance inflation factors for explanatory variables were very close to 1 (1.07 for both explanatory variables: tag burden and body condition), indicating that variables included in the model were not strongly correlated.

The significance of each explanatory variable was tested using likelihood ratio tests between nested models in the *lmtest* package for R (Zeileis & Hothorn, 2002), with insignificant variables removed in a step‐down approach until a final model was selected. For all models, model residuals were checked using the *DHARMa* package (Hartig, 2022) in R to determine whether model assumptions were met.

#### 2.6.2 Spatial and temporal variations in migration speed

Duration and speed of migration were calculated for each individual smolt. Duration was defined as the total time difference between the last detection at the first acoustic receiver to the first detection at the last acoustic receiver in the river. Migration speed was defined as the total river distance traveled divided by duration and expressed as kilometers per day (km day^−1^).

Generalized linear models with a gamma distribution were used to test whether migration speed (km day^−1^) showed significant differences between rivers or years, testing the use of each of these explanatory variables against a null model of the intercept using likelihood ratio tests.

#### 2.6.3 Population migration success

Migration success was also calculated at the population level (i.e., for each river). Population migration success was defined as the number of tagged salmon smolts detected at the last most downstream receiver expressed as a proportion of all smolts that were detected passing the first most upstream receiver. Thus, smolts detected at the final freshwater receiver were deemed to have undertaken a successful river migration.

A minimum estimate of detection efficiency of the first receiver in each river for each year was calculated as the number of fish detected on the first receiver divided by the number of fish known to have passed the first receiver (i.e., the sum of the number of fish detected on the first receiver and the number of fish “missed” by the first receiver but detected on the second receiver). Where possible, detection efficiency of the second, most downstream receiver was determined using the same process but using data from marine receivers, which was possible for only eight rivers (rivers Deveron, Spey, Findhorn, Ness, Conon, Oykel, Shin, and Orchy). For the eight rivers where detection efficiency of the last receiver was measured, the mean efficiency was 91.3%. Thus, this measure of river migration success must be regarded as a minimum estimation of the success of river migration as it is possible that some smolts could have left the system undetected. However, the relatively high overall detection efficiency, where it was measured, does suggest that relatively few tagged fish passed undetected.

#### 2.6.4 Spatial variation in population migration success

To determine whether there was significant spatial variation in migration success, a general linear model was constructed with migration success as the response variable and river as a categorial explanatory variable, the significance of which was tested against a null model of the intercept using a likelihood ratio test.

#### 2.6.5 Temporal variation in population migration success

To test whether migration success significantly varied over time, only rivers with >1 year of migration data (*N* = 13) were included in this analysis. A χ^2^ goodness‐of‐fit test was used to determine whether the proportion of successful migrants significantly differed in subsequent years from the proportion of fish making a successful migration in the first year. To test whether temporal variation in migration success was consistent across rivers, Kendall's coefficient of concordance was calculated for migration success in each year of the study across each river.

For all further analysis, the rate of migration success was used. This metric is the mean, for each river, of the proportion of all fish successfully migrating over each river kilometer as a proportion of those at the start of that kilometer. It was calculated using the following formula:

Rate of migration success = overall population migration success^1/river distance^ (expressed as proportion km^−1^).

Finally, to test whether within‐river variation in rate of migration success differed across rivers, a Levene's test was used to test for similarity of variance in the *car* package in R (Fox & Weisberg, 2019).

#### 2.6.6 Environmental determinants of spatial variation in population migration success

To determine whether spatial variation in the rate of migration success (proportion km^−1^) could be predicted by environmental variables, a generalized linear model with a beta distribution and a logit link function was developed using the *glmmTMB* package (Brooks et al., 2017). Rate of migration success was used as the response variable, and a top‐down model selection approach using likelihood ratio tests (Zeileis & Hothorn, 2002) was used to determine the significance of the explanatory variables tested. Continuous explanatory variables included in the initial model were catchment size (km^2^); the proportion of five land cover types in the catchment (grassland, woodland, wetland, urban areas, and agricultural land); the proportion of the catchment's superficial geology comprising peat; catchment gradient (m m^−1^.100); and the number of barriers in the river channel. In addition, the dominant bedrock type in the catchment was included as a categorical explanatory variable with three levels (igneous, metamorphic, and sedimentary).

The *DHARMa* package (Hartig, 2022) was used to test and confirm that the model met assumptions of independence and homoscedasticity of residuals. The model was also checked for overfitting by cross‐validation using the *cv* package (Fox & Monette, 2024). Tukey's adjusted pair‐wise comparisons of estimated marginal means were computed using the *emmeans* package (Lenth, 2023) to determine the relationship between each level of the categorical variables with the response variable and the significance of that relationship. The final model was used to generate predicted values of the response variable across the observed range of each explanatory variable while holding other explanatory variables constant. For the purposes of visualization only, these predictions, representing the marginal effects of each explanatory variable, were generated as a rate of migration success standardized for migration distance. To standardize for migration distance, we used the mean river migration distance of all rivers in the study area (31.54 km). Results were visualized using *ggplot2* (Wickham, 2016).

#### 2.6.7 Environmental determinants of temporal variation in population migration success

To test whether a standardized measure of river discharge (percentage exceedance) predicted rate of migration success, a generalized linear mixed model with a beta distribution and a logit link function was constructed. Rate of migration success was used as the response variable, percentage exceedance as a continuous explanatory variable, and river as a random effect. Only rivers that had >1 year of data were used in this model (*n* = 13). The significance of the effect of percentage exceedance was tested using a likelihood ratio test against a nested null model without percentage exceedance as an explanatory variable.

## 3 RESULTS

A total of 4120 salmon smolts were tagged between 2019 and 2022. With the exception of two outliers, the detection efficiency of the first receivers in each river ranged from 62.5% to 100%, with a mean detection efficiency of 94.5% (±10.0% SD). The two outliers were the first receiver in the River Gryffe in 2022, which had 0% detection efficiency, and the first receiver in the River Deveron in 2021, which also had very low detection efficiency (2.7%). Consequently, fish were deemed to have entered the study at the release site rather than arrival at the first receiver for the rivers Deveron (in 2021) and Gryffe (in 2022).

### 3.1 Determinants of river migration success in individual fish

Using a generalized linear mixed effects model, with binomial distribution, migration success of individual fish from across all rivers and all years was significantly predicted by tag burden (2ΔLL = 4.2, *df* = 1, *p* = 0.029) and body condition (2ΔLL = 4.2, *df* = 1, *p* = 0.009). The model‐predicted probability of migration success decreased from 0.49 to 0.27 as tag burden increased from 1.00% (the minimum tag burden in this study) to a maximum of 9.15%. The predicted probability of migration success increased from 0.12 to 0.56 as body condition increased from the minimum observed value in this study (−1.44) to its maximum of 0.57.

### 3.2 Migration speed

The slowest mean migration speed observed was in the River Deveron in 2022 (1.64 km day^−1^), whereas the highest mean migration speed occurred in the River Findhorn in 2021 (58.98 km day^−1^) (Table 2). Using a generalized linear model with a gamma distribution, speed of migration differed significantly between rivers (2ΔLL = 78.15, *df* = 22, *p* < 0.001) but not between years (2ΔLL = 3.98, *df* = 3, *p* = 0.27).

**TABLE 2:** River migration success (the number of individuals detected exiting the river as a proportion of fish detected on the first most upstream receiver), rate of migration success (migration success standardized for river length [proportion km^−1^]), and mean migration speed (km day^−1^ ± SD) for each river and each year.

River	Year	Migration success	Rate of migration success (proportion km^−1^)	Mean speed (km day^−1^ ± SD)
Aberdeenshire Dee	2019	0.645	0.996	8.59 ± 3.56
Agivey	2021	0.657	0.984	7.23 ± 5.00
Bladnoch	2021	0.730	0.903	4.76 ± 9.39
Bladnoch	2022	0.761	0.949	12.24 ± 10.00
Burrishoole	2021	0.821	0.941	13.93 ± 27.26
Bush	2019	0.804	0.911	17.69 ± 14.64
Bush	2020	0.909	0.960	14.20 ± 11.16
Bush	2021	0.961	0.983	10.47 ± 10.01
Bush	2022	0.968	0.986	9.18 ± 10.30
Cassley	2021	0.682	0.990	3.38 ± 1.33
Conon	2019	0.902	0.982	16.60 ± 26.62
Conon	2021	0.647	0.964	9.25 ± 18.36
Crawick	2021	0.774	0.994	10.86 ± 9.29
Derwent (released at tagging site)	2020	0.313	0.976	7.09 ± 3.57
Derwent (released at tagging site)	2021	0.327	0.977	3.31 ± 1.53
Derwent (transported)	2021	0.718	0.986	5.53 ± 3.94
Derwent (transported)	2022	0.515	0.972	3.31 ± 2.61
Deveron	2019	0.514	0.989	4.65 ± 1.72
Deveron	2021	0.740	0.996	7.03 ± 3.13
Deveron	2022	0.115	0.974	1.64 ± 0.0
Endrick	2020	0.333	0.968	3.05 ± 1.98
Endrick	2021	0.135	0.942	3.03 ± 1.36
Findhorn	2019	0.438	0.911	9.36 ± 9.91
Findhorn	2021	0.863	0.972	58.98 ± 43.83
Kirkcudbrightshire Dee	2021	0.225	0.968	3.02 ± 0.89
Gryffe	2021	0.929	0.989	3.15 ± 3.90
Gryffe	2022	0.837	0.979	1.99 ± 1.49
Leven	2021	0.377	0.750	15.92 ± 13.66
Leven	2022	0.889	0.989	8.76 ± 12.28
Ness	2019	0.102	0.959	4.42 ± 2.05
Ness	2021	0.198	0.972	2.79 ± 1.05
Ness	2022	0.625	0.988	3.77 ± 2.93
Nith	2021	0.825	0.985	10.72 ± 21.14
Nith	2022	0.742	0.987	5.24 ± 3.57
Orchy	2022	0.375	0.847	4.40 ± 4.72
Oykel	2019	0.704	0.989	5.97 ± 2.94
Oykel	2021	0.841	0.994	4.72 ± 1.88
Oykel	2022	0.468	0.975	5.27 ± 2.67
Scuar	2022	0.632	0.986	4.96 ± 2.95
Shin	2019	0.918	0.992	2.76 ± 6.50
Shin	2021	0.823	0.982	2.21 ± 6.45
Spey	2019	0.610	0.990	7.03 ± 3.33
Spey	2021	0.411	0.980	9.74 ± 5.36
Spey	2022	0.034	0.972	6.68 ± 2.12

### 3.3 Spatial variation in population migration success

Population migration success varied between rivers, from 0.03 (River Spey, in 2022) to 0.97 (River Bush, in 2022) (Table 2). Tested for using a general linear model, migration success showed significant spatial variation (2ΔLL = 52.14, *df* = 22, *p* < 0.001).

### 3.4 Environmental determinants of spatial variation in population migration success

The rate of river migration success of populations was significantly predicted by a number of catchment characteristics. This was tested for using a generalized linear model with a beta distribution and a logit link function. Thus, the proportion of grassland (*m*_1_), woodland (*m*_2_), wetland (*m*_3_), and peat (*m*_4_) in the catchment, as well as bedrock type (*α*_*j*_), significantly predicted migration success:

$$\begin{array}{l} F_{i}=m_{1}\cdot x_{1,i}+m_{2}\cdot x_{2,i}+m_{3}\cdot x_{3,i}+m_{4}\cdot x_{4,i}+\alpha_{j};i=1,\ldots 23,\\ j=\text{\{igneous, metamorphic, sedimentary\}.} \end{array}$$

Explanatory variables that were not included in the final model were the proportions of the catchment comprising agricultural land and urban land, catchment size, river gradient, and the number of barriers in the river. To test for unwanted and disproportionate effects of a small number of rivers in driving these effects, the two rivers with most extreme values of these variables were removed, and the final model was rerun. The overall pattern of explanatory variable inclusion did not change by doing this.

**FIGURE 2:**
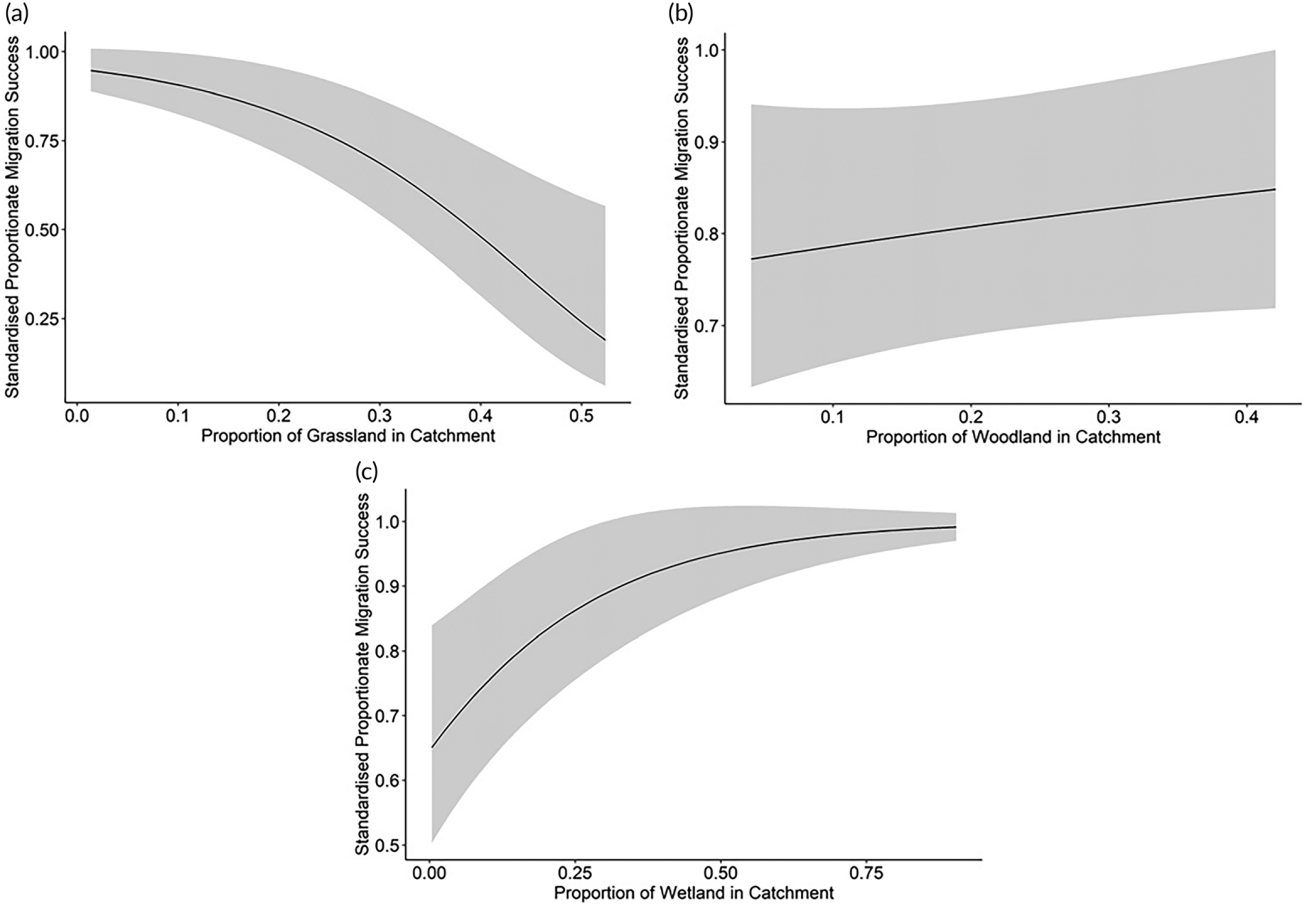
The model predicted relationships between the marginal effects of three land cover variables on standardized proportionate migration success, generated using a beta‐distributed GLM (generalized linear model). Standardized rate of migration success is the river‐specific rate of migration success standardized to a river length of 31.52 km (the mean river length across all study sites). Solid black line shows predicted values, with the gray ribbon showing a confidence interval of 2 SDs on either side of the mean. Predicted values of standardized rate of migration success against (a) the proportion of grassland in catchment, (b) the proportion of woodland in catchment, and (c) the proportion of wetland in catchment.

The proportion of grassland cover in the catchment had a negative effect on the rate of migration success (parameter estimate = −6.74; 2ΔLL = 18.84, *df* = 1, *p* < 0.001). Model‐predicted standardized rate of migration success (standardized to a mean river distance of 31.52 km) decreased from 0.947 for catchments with the lowest observed proportion of grassland to 0.189 for catchments with the highest observed proportion of grassland (Figure 2a). The decrease in the standardized rate of migration success was non‐linear, becoming much steeper as the proportion of grassland increased beyond ~0.2 (Figure 2a).

There was a marginally significant positive effect of the proportion of woodland cover in the catchment on the rate of migration success (parameter estimate = 1.19; 2ΔLL = 3.07, *df* = 1, *p* = 0.0797). The model predicted a standardized rate of migration success of 0.772 for catchments with the lowest proportion of woodland cover (0.041) compared to 0.848 for the highest (0.421) in this study (Figure 2b).

The proportion of the catchment comprising wetland had a positive effect on the rate of migration success (parameter estimate = 4.35; 2ΔLL = 9.60, *df* = 1, *p* = 0.002). This effect was non‐linear with a stronger effect at lower values of wetland cover, with the standardized rate of migration success increasing more rapidly as proportionate wetland cover increased from 0 to 0.25. When the standardized rate of migration success increased above 0.25, the effect of wetland cover was less marked (Figure 2c). Overall, the model predicts the standardized rate of migration success increasing from 0.651 for the lowest observed proportion of wetland cover in a catchment (0.0039) to 0.991 at the highest proportion of wetland cover (0.904) (Figure 2c).

**FIGURE 3:**
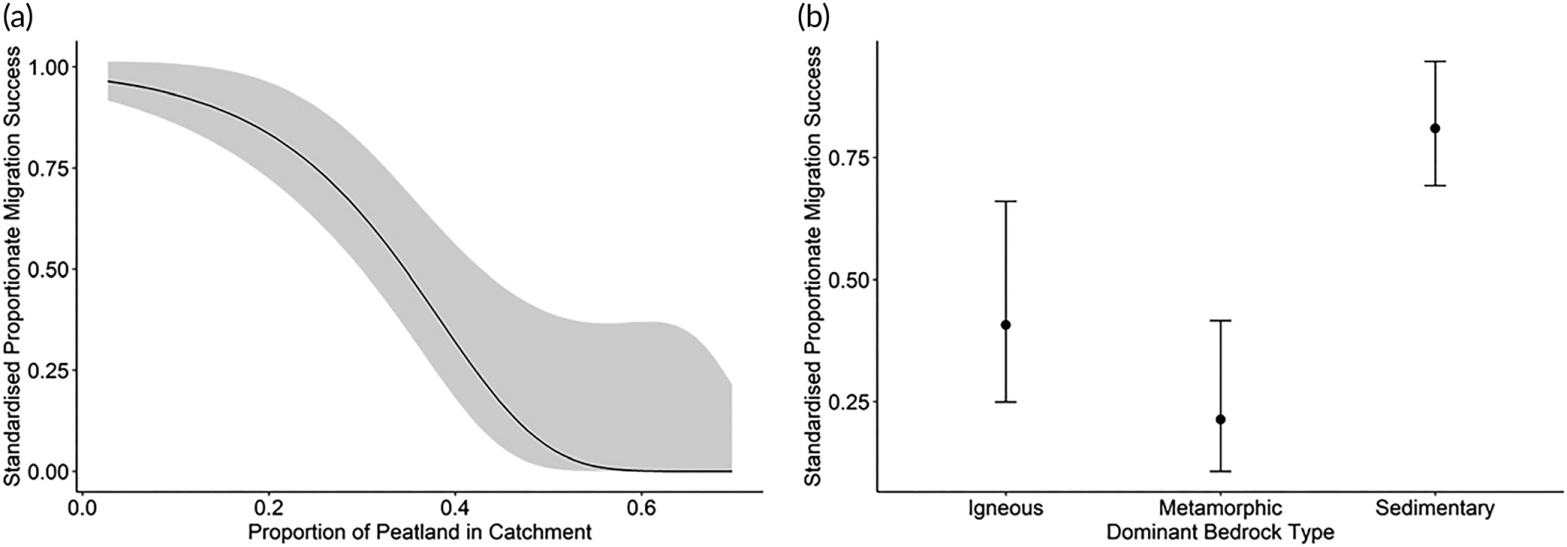
The model predicted marginal effects of two geological explanatory variables on standardized rate of migration success, generated using a beta‐distributed GLM (generalized linear model). Standardized rate of migration success is the river‐specific rate of migration success applied to a standard river length of 31.52 km (mean river length across study sites). (a) Predicted values of standardized rate of migration success against the proportion of peat in the superficial geology of the catchment. Solid black line shows predicted values, with gray ribbon showing a confidence interval of 2 SDs on either side of the mean. (b) The model predicted the relationship between standardized rate of migration success and each of the three bedrock categories: igneous, metamorphic, and sedimentary. The black points show the predicted mean values for each bedrock category, with confidence intervals showing 2 SEs around this mean.

The model found significant effects of both superficial soil type and bedrock geology on the rate of migration success. The proportion of peatland strongly and negatively predicted rate of migration success in this study (parameter estimate = −9.29; 2ΔLL = 15.15, *df* = 1, *p* < 0.001). The explanatory model predicted a standardized rate of migration success of <0.0001 for the catchments with the highest proportion of peat cover measured in this study (0.70) compared to 0.964 for the lowest (0.027) (Figure 3a).

The dominant bedrock type in the catchment also predicted the rate of migration success (parameter estimates: igneous = 6.02, metamorphic = −0.55, sedimentary = 1.46; 2ΔLL = 14.56, *df* = 2, *p* < 0.001). Standardized rate of migration success in catchments dominated by sedimentary rock was significantly higher (mean ± SD = 0.810 ± 0.07) than those catchments dominated by igneous (0.407 ± 0.13) or metamorphic rock (0.213 ± 0.10) (Tukey's HSD [honestly significant difference], *p* < 0.001). However, there was no significant difference in standardized rate of migration success between catchments dominated by igneous and metamorphic rocks (Tukey's HSD, *p* = 0.38) (Figure 3b).

### 3.5 Temporal variation in population migration success

For those rivers for which migration success was measured >1 year (*N* = 13), there was also significant temporal variation (tested for using χ^2^ goodness‐of‐fit test). Thus, for these rivers, the frequency of fish migrating successfully in subsequent years differed significantly from that of the first year (χ^2^ = 365.50, *df* = 20, *p* < 0.001) (Table 2). The greatest difference in migration success measured in this study was in the River Spey, where the difference in the proportion of fish migrating successfully between the first and last years of measurement (2019 and 2022) was 0.576.

Results from the Kendall's coefficient of concordance demonstrated that the pattern of temporal variation in migration success was not consistent across the rivers included in this study (*W* = 0.60, *df* = 13, *p* = 0.12). A Levene's test of similarity of variance showed that variation in the rate of migration success between years, a measure of the stability of the migration success for each river, differed between rivers (*F*_14,21_ = 17.46, *p* < 0.001) (Table 3). Thus, in some rivers migration success was more temporally stable than in others.

**TABLE 3:** A measure of the temporal stability of the rate of migration success, measured across all years for rivers with >1 year of data.

River	Maximum range of standardized rate of migration success
Bladnoch	0.0469
Bush	0.0261
Conon	0.0181
Derwent (released at tagging site)	0.0016
Derwent (transported)	0.0137
Deveron	0.0222
Endrick	0.0256
Findhorn	0.0614
Gryfe	0.0103
Leven	0.2383
Ness	0.0290
Nith	0.0025
Oykel	0.0189
Shin	0.0010
Spey	0.0180

### 3.6 Environmental determinants of temporal variation in population migration success

Using a generalized linear mixed model with a beta distribution and a logit link function, the variation in the rate of migration success (with river as a random factor) was not predicted by a measure of river discharge standardized for river size (exceedance percentage) (2ΔLL = –1.04, *df* = 1, *p* = 0.31).

## 4 DISCUSSION

In part, because of the logistical burden, as well as the expense of conducting telemetry studies in general, most studies on the migration of Atlantic salmon smolts as they migrate to sea have been conducted on one or two rivers and typically over a single year (however, see Chaput et al., 2019). In the study presented here, we used telemetry data from 22 different rivers in which salmon smolts were tracked in the same year. Of these, 13 rivers provided data from multiple years. This approach provides for a powerful study design where we are able to show that the downstream river migration success of Atlantic salmon smolts varies both spatially (across rivers) and temporally (among years). The magnitude of variation in migration success is considerable, with migration success ranging from 3.4% (the River Spey) to 97.0% (the River Bush) between rivers and from 3.4% to 61.0% over time in the same river (over 3 years in the River Spey). In this study, migration success is defined as the number of smolts that were detected on the most downstream acoustic receiver expressed as a proportion of those that were detected on the most upstream receiver. It is unlikely that all downstream acoustic receivers had a detection efficiency of 100%, and as such, fish may have migrated past the receiver undetected. Therefore, it is the minimum migration success that is being measured here. Although in this study we were unable to assess the detection efficiency of all the final downstream receivers, the mean of those from eight rivers (91%) suggests that there were relatively few detections missed by the last river receiver.

One likely driver of the high temporal variation in migration success between years is the broad‐scale weather patterns. For example, Michel et al. (2021) found a strong influence of river flow rate on the migration success of Chinook salmon (*Oncorhynchus tshawytscha*). Similarly, both Flávio et al. (2020) and Gauld et al. (2013) showed reduced migration success for Atlantic salmon smolts during years with low river discharge compared with those with high discharge. Temporal variation in migration success in Atlantic salmon has been demonstrated elsewhere (see Chaput et al., 2019; Flávio et al., 2021; Stich et al., 2015). However, the results of the study presented here show that temporal variation in migration success was not consistent between rivers and instead varied on a river‐by‐river basis. For example, the River Bush showed more stable migration success across years (migration success ranged from 80% to 97% over 4 years) in contrast to the River Spey (migration success ranged from 3.4% to 61%). Thus, this would suggest that the potential environmental drivers of variation in migration success are acting at a local catchment scale rather than being driven by environmental variables operating at a much larger spatial scale (e.g., broad interannual weather patterns). In addition, this study strongly points toward some rivers and their populations having more stable patterns of migration success than others. We were unable to determine from this study what extrinsic or intrinsic river or population factors might influence the stability (or lack of it) of salmon smolt migration success, but it is a potential area for future investigation.

### 4.1 Individual migration success

The probability of an individual making a successful river migration across all study sites and all years was predicted by body condition and tag burden. Body condition (where values comprise fish with a greater mass independent of body length) positively predicted migration success and had a predicted 4.7‐fold effect on the likelihood of a fish undertaking a successful migration across the range in fish in this study. Previous studies have examined the role of body condition in migration success with varying results. Some studies have found no relationship between migration success and body condition (e.g., see Lilly et al., 2022), whereas others have shown it to be important in predicting migration success (e.g., Antonsson et al., 2010; Armstrong et al., 2018). However, where it has been detected previously, this tends to have been found as a relationship between smolt body condition and the probability of returning as adults.

The tag burden to which individual fish were exposed also affected the probability of river migration success. This effect was much smaller than that for body condition, with the magnitude of the model predicted effect being 1.8‐fold over the range in this study. The tag burden to which fish are exposed in telemetry studies has been a contentious issue that has been investigated through empirical studies in recent years. Studies have shown no elevated mortalities or tag‐related mortalities at tag burdens ranging from 2.9% (Moore et al., 1990) to 12.7% (Brunsdon et al., 2019). A number of other studies have also looked for tag burden effects of acoustic telemetry tags on migration success and failed to find an effect (Daniels et al., 2021; Lennox et al., 2022; Lilly et al., 2022; Lothian et al., 2018; Newton et al., 2016). In contrast, here we show that tag burden over the range of 1% to 9.15% did significantly affect migration success of individual smolts. It is possible that the very large sample size provided in the study presented here has the power to detect such an effect where others do not. It is reasonable to suggest that the mechanism through which landscape variables have an effect on migration success might be mediated through their effects on the body condition of individual fish from that river. However, in our dataset, as the significant landscape variables identified here were not significantly correlated with either tag burden or condition factor, this suggests that this was not the mode of effect in this study.

### 4.2 Environmental determinants of spatial variation in population migration success

In this study we found that the proportion of downstream migrants that made a successful migration was significantly predicted by a broad suite of catchment landscape and river characteristics through which the smolts were migrating. Broadly, these environmental predictors can be separated into natural environmental catchment features, such as underlying bedrock type, and environmental features that could be, or have been, altered by human activities, including the proportions of the catchment that comprise wetland, grassland, and woodland and the proportion of peat in the catchment. Although in this study we identify some specific landscape features that significantly predict migration success, it is very possible that for other river types with characteristics differing from those included here, different landscape features may influence migration success. We suggest that further testing of this effect in different catchments is an important area for future study.

An analysis of the detailed mechanisms through which these characteristics influence smolt migration success is beyond the scope of the study presented here; however, there are some logical inferences that provide some insights into these mechanisms of effect. Migration success of salmon smolts was significantly and negatively predicted by the proportion of peat in a catchment. Peatland has become highly degraded in the United Kingdom and Ireland by human activity. It is often either drained for agriculture or exploited for the peat reserves (Minasny et al., 2023). One possible mechanism through which salmon migration success might be negatively affected by the proportion of peat in a catchment is through drainage. Peatland water runoff is acidic in nature and in combination with other anthropogenic activities, such as conifer plantations, can lead to river channel acidification that has the potential to negatively impact on the condition of salmonids (Saarinen et al., 2013).

The proportion of woodland in the catchment had a small and marginally significant but positive effect on smolt migration success. Woodland can moderate river flow by slowing the speed of drainage of precipitation into the river channel, thus reducing the magnitude of flow peaks and troughs (Revell et al., 2021). It is thus possible that the effect of woodland on migration success is modulated through the hydrological effects on rivers, allowing longer flow windows for successful migration. In addition, riparian forest cover has been shown to moderate the thermal regime of rivers during the summer (Hrachowitz et al., 2010; Jackson et al., 2021). Thus, it is possible that thermal stress could negatively affect smolts during their downstream migration (McCormick et al., 1999). However, there are other possible mechanisms. High levels of riparian cover may mitigate against avian predation, which is known to be relatively high in some rivers (Chavarie et al., 2022). Riparian cover is associated with higher macroinvertebrate densities in some catchment types (Palt et al., 2023). Thus, it is possible that this effect impacts migration success through improved condition of salmon smolts in rivers with high proportions of woodland in the catchment.

Higher proportions of wetlands in the catchment resulted in greater migration success. Catchments with a higher proportion of wetlands are usually indicative of more naturally draining river systems with a hydrology that is more buffered from rapid fluctuations in flow. Thus, although speculative, it is possible that, as with the effect of woodlands, wetlands buffer river flow extremes.

The proportion of the catchment that comprised grassland had a negative effect on migration success. In the United Kingdom and Ireland grassland is mostly a human‐modified habitat type resulting from current or ancient agriculture. Grassland is likely to be better drained and have a lower rainwater interception capacity compared with, for example, woodland, which had the opposite effect on salmon migration (Revell et al., 2021). Thus again, although speculative, the effect of grassland on migration success may be mediated through river water flow rates.

Finally, smolt migration success was influenced by the relative proportion of bedrock type in the catchment. Fish from rivers dominated by sedimentary rocks were more likely to have successfully migrated than those catchments dominated by igneous or metamorphic geologies. Rivers running through sedimentary geologies are more likely to be nutrient rich than those dominated by igneous or metamorphic rock types. It is thus possible that the natural baseline nutrient loading of such rivers impacts smolts through nutrition and has an impact on migration success through that mechanism. However, as with the other mechanisms articulated here, the study provides evidence of which catchment and individual fish characteristics influence salmon smolt migration success but does not provide any direct evidence of the mechanisms underlying these effects.

## 5 CONCLUSION

This study provides previously unknown but valuable insights into some of the landscape‐scale environmental drivers of downstream migration success in Atlantic salmon smolts at a population level. These insights require further investigation to determine the underlying mechanisms through which these environmental factors act, but irrespective of this, this study provides important information for the effective management of the species at a catchment scale. Although the environmental drivers of the temporal variation in migration success were not identified in this study, we have shown that environmental factors are likely mostly catchment specific rather than at larger geographic scales, as temporal variation in migration success was not consistent across rivers.

## AUTHOR CONTRIBUTIONS

Jessica R. Rodger, Colin E. Adams, Angus J. Lothian, and Hannele M. Honkanen designed and planned this study; Jessica R. Rodger, Hannele M. Honkanen, Angus J. Lothian, Jessie Lilly, Marcus Walters, Richie Miller, Lorraine Hawkins, Al Reeve, Jamie Ribbens, Jim Henderson, Debbie Parke, Amy Green, Alan Kettle‐White, Brian Shaw, Stephen Burns, Robert Laughton, Chris Conroy, Chris Daphne, Keith Williams, Sean Robertson, Catherine Waters, Deirdre Cotter, Niall Ó Maoiléidigh, and Richard Kennedy executed the fieldwork for the nine projects from which the data were collated from for this study; Jemma Guthrie analysed the data; Jessica R. Rodger, Colin E. Adams, and Jemma Guthrie drafted the manuscript with feedback from all authors.

## FUNDING INFORMATION

This study was funded by the Atlantic Salmon Trust, EU award IVA5060 from the European Union, Interreg 5A programme, the Moray Firth project, Salmon Scotland, the Maritime Fisheries Fund, the Environment Agency, Natural England, the Derwent Owners Association, United Utilities PLC, NatureScot, the Nith District Salmon Fishery Board, the Holywood Trust, Dumfries and Galloway Council, the Missing Salmon Alliance, Drax, the Dee (Kirkcudbrightshire) District Salmon Fishery Board, and the Galloway Glens Landscape Partnership Project.

## Supporting information


**Table S1.** Latitude and longitude of each receiver deployed in each year. Only two receivers were deployed in each river each year: receiver 1, which was the most upstream receiver, was positioned to detect fish entering the study system and receiver 2, which was the most downstream receiver, was positioned to detect fish exiting the river system.
**Table S2.** Specifications of acoustic tags used in this study. All tags were supplied by Innovasea, Canada.

## Data Availability

Data will be made available on reasonable request to the authors.
