# Supplementary material for: Determinants of riverine migration success by Atlantic salmon (Salmo salar) smolts from rivers across the UK and Ireland
Source: J Fish Biol. 2024 Aug 12;106(5):1585–600. doi: 10.1111/jfb.15884 (PMC12120332; doi:10.1111/jfb.15884)
Supplement: Supplementary file 1 — Table S1. Latitude and longitude of each receiver deployed in each year. Only two receivers were deployed in each river each year: receiver 1, which was the most upstream receiver, was positioned to detect fish entering the study system and receiver 2, which was the most downstream receiver, was positioned to detect fish exiting the river system. Table S2. Specifications of acoustic tags used in this study. All tags were supplied by Innovasea, Canada. [file JFB-106-1585-s001.docx]

*Table S1 Latitude and longitude of each receiver deployed in each year. Only two receivers were deployed each river each year, receiver 1 which was the most upstream receiver and was positioned to detect fish entering the study system and receiver 2 which was the most downstream receiver and was positioned to detected fish exiting the river system.*

|  |  | **Receiver 1** | | **Receiver 2** | |
| --- | --- | --- | --- | --- | --- |
| **River** | **Year** | **Latitude** | **Longitude** | **Latitude** | **Longitude** |
| Aberdeenshire Dee | 2019 | 56.93567 | -3.4155 | 57.14453 | -2.06326 |
| Agivey | 2021 | 55.01134 | -6.6568 | 55.15348 | -6.69671 |
| Bladnoch | 2021 / 2022 | 54.86528 | -4.48884 | 54.85591 | -4.4512 |
| Burrishoole | 2021 | 53.92163 | -9.57135 | 53.89821 | -9.57563 |
| Bush | 2019 – 2022 | 55.21052 | -6.52663 | 55.21924 | -6.53367 |
| Cassley | 2021 | 58.1424 | -4.7787 | 57.92259 | -4.40003 |
| Conon | 2019 / 2021 | 57.55138 | -4.49369 | 57.57345 | -4.43466 |
| Crawick | 2021 | 55.34003 | -3.84883 | 55.04566 | -3.60673 |
| Derwent (released at trapping site) | 2020 / 2021 | 54.61388 | -3.06569 | 54.64946 | -3.5238 |
| Derwent (transported) | 2021 / 2022 | 54.68688 | -3.30222 | 54.64649 | -3.54242 |
| Deveron | 2019 / 2021 - 2022 | 57.46305 | -2.85291 | 57.65907 | -2.51263 |
| Endrick | 2020 - 2021 | 56.05136 | -4.447 | 55.93811 | -4.56415 |
| Findhorn | 2019 | 57.6031 | -3.65323 | 57.66033 | -3.62752 |
| Findhorn | 2021 | 57.59822 | -3.65372 | 57.63313 | -3.63723 |
| Kirkcudbrightshire Dee | 2021 | 55.13042 | -4.18351 | 54.8313 | -4.06791 |
| Gryffe | 2021 / 2022 | 55.8746 | -4.48258 | 55.89042 | -4.40565 |
| Leven | 2021 / 2022 | 55.96273 | -4.57785 | 55.93867 | -4.56333 |
| Ness | 2019 | 57.07238 | -4.76978 | 57.45355 | -4.26146 |
| Ness | 2021 | 57.07222 | -4.76972 | 57.47116 | -4.26055 |
| Ness | 2022 | 57.09874 | -4.7407 | 57.45249 | -4.26167 |
| Nith | 2021 | 55.12133 | -3.63899 | 55.04566 | -3.60673 |
| Nith | 2022 | 55.14827 | -3.68779 | 55.0043 | -3.57613 |
| Orchy | 2022 | 56.40833 | -5.16754 | 56.4418 | -5.22945 |
| Oykel | 2019 / 2021 - 2022 | 57.99353 | -4.80004 | 57.92259 | -4.40003 |
| Scuar | 2022 | 55.21325 | -3.76846 | 55.0043 | -3.57613 |
| Shin | 2019 / 2021 | 58.00536 | -4.4036 | 57.92259 | -4.40003 |
| Spey | 2019 | 57.42069 | -3.37551 | 57.67419 | -3.0967 |
| Spey | 2021 | 57.42066 | -3.37559 | 57.63862 | -3.09925 |
| Spey | 2022 | 57.08547 | -4.01352 | 57.67442 | -3.09756 |

*Table S2 Specifications of acoustic tags used in this study. All tags were supplied by Innovasea, Canada*

| **Tag type** | **Transmission interval range (seconds)** | **Tag diameter/length (mm)** | **Tag weight in air (g)** | **Power output (dB re 1μPa @ 1m)** |
| --- | --- | --- | --- | --- |
| V5-1x | 20-40 | 4.3 x 5.73 / 12.7 | 0.64 | 141 |
| V6-2x | 18-38 | 6.3/13 | 0.9 | 137 |
| V7-2x | 18-60 | 7/19.5 | 1.5 | 137 |
| V7-4x | 20-60 | 7/21.5 | 1.8 | 137 |
| V7D-2x | 15-35 | 7/21.5 | 1.8 | 137 |
| V8-4x | 40-80 | 8/20.5 | 2.0 | 144 |
